# Caki-1 Spheroids as a Renal Model for Studying Free Fatty Acid-Induced Lipotoxicity

**DOI:** 10.3390/cells14050349

**Published:** 2025-02-27

**Authors:** Dana Battle, Xiangzhe Qiu, Marilyn Alex, London Rivers, Jamie A. G. Hamilton, Shuichi Takayama, Xueying Zhao

**Affiliations:** 1 Department of Physiology, Morehouse School of Medicine, Atlanta, GA 30310, USA; dbattle@msm.edu (D.B.); tom3635qiu@gmail.com (X.Q.); marilynalexg@gmail.com (M.A.); london3rivers@gmail.com (L.R.); 2 Wallace H. Coulter Department of Biomedical Engineering, Georgia Institute of Technology and Emory University School of Medicine, Atlanta, GA 30332, USA; jamie.hamilton@bme.gatech.edu (J.A.G.H.); takayama@gatech.edu (S.T.); 3 The Parker H. Petit Institute of Bioengineering and Bioscience, Georgia Institute of Technology, Atlanta, GA 30332, USA

**Keywords:** lipotoxicity, lipid droplet, autophagy, ER stress, tubular epithelial cells

## Abstract

Lipotoxicity, resulting from the buildup of excess lipids in non-adipose tissues, is increasingly recognized as a major contributor to the progression of kidney disease, highlighting the need for alternative models to assess its effects on renal cells. The main aim of this study was to investigate the usefulness of Caki-1, a human proximal tubule (PT) and renal cell carcinoma (RCC) representative cell line, as a 3D model system for studying free fatty acid-induced PT lipotoxicity. Caki-1 spheroids were generated and maintained on ultra-low attachment plates and characterized regarding time-dependent morphology changes. In optimal 3D culture conditions, Caki-1 cells formed well-defined large compact spheroids with uniform morphology, good circularity, and increased diameter from days 4–12. Chronic exposure to saturated palmitate resulted in dose- and time-dependent spheroid disintegration and cell death, including dispersed and flattened spheroid morphology, with increased dead cells in the peripheral layers and decreased spheroid core. Moreover, palmitate-treated spheroids showed a significant increase in cleaved poly(ADP-ribose) polymerase (PARP) and active caspase-3. Palmitate-induced PARP cleavage, as well as endoplasmic reticulum (ER) stress and autophagy dysfunction, were blunted by triacsin C, an inhibitor of long-chain acyl-CoA synthetases. In addition, co-incubation with unsaturated oleate prevented palmitate-induced spheroid disintegration and apoptotic cell death in Caki-1 3D culture. While fatty acid overload upregulated lipid droplet protein perilipin 2 in Caki-1 cells, knockdown of perilipin 2 by siRNAs resulted in an exacerbation of palmitate-induced cell death. Together, these results indicate that the 3D Caki-1 spheroid model is a simple and reproducible in vitro system for studying renal lipotoxicity and lipid metabolism that gives useful readouts at the molecular, cellular, and multicellular levels.

## 1. Introduction

Chronic kidney disease (CKD), characterized by long-term functional decline and progressive structural damage in the kidneys, is increasing globally and affects approximately 850 million people worldwide [1]. Moreover, the prevalence of kidney disease and renal cancers increases further due to the obesity epidemic and environmental changes, causing more serious implications for survival, quality of life, and health care spending. Current treatment for CKD focuses on slowing the progression of kidney damage, usually by controlling the cause (e.g., strict glycemic and blood pressure control) [2,3,4]. However, even controlling the cause might not halt the progression of CKD to end-stage kidney disease (ESKD), which requires dialysis or kidney transplantation.

Proteinuria, a biomarker and risk predictor for CKD-associated complications, has also been recognized as a driver of CKD progression toward ESKD [5,6]. Increasing evidence points to a tight association of CKD progression with tubulointerstitial damage due to the cytotoxicity of filtered urinary proteins to proximal tubule epithelial cells (PTECs), regardless of the causes of proteinuria. Importantly, the damaged glomerulus allows albumin-bound free fatty acids (FFAs) to be filtered and accumulated predominantly in PTECs [7,8,9,10,11]. Over-reabsorption of palmitate, the most abundant saturated FFA in human blood and urine, leads to intracellular lipid accumulation and lipotoxicity, including endoplasmic reticulum (ER) stress, autophagy dysfunction, and apoptosis [10,11,12]. In contrast, oleate, the most unsaturated FFA, does not cause toxic effects and even prevents palmitate-induced renal lipotoxicity [10]. Recent studies further demonstrate that oleate overload promotes the accumulation of lipid droplets (LDs), which provide a dynamic lipid repository that can safely sequester lipids to prevent lipotoxicity and can be mobilized upon cellular demand to provide substrates for the generation of energy (i.e., β-oxidation) or the biosynthesis of lipid signaling molecules and membranes [10,13,14].

While in vitro cell-based models utilizing PTECs are widely used for renal cytotoxic research, previous lipotoxic studies largely rely on two-dimensional (2D) kidney cell culture due to its simplicity, cost-effectiveness, and repeatability. Recently, 3D cell culture models, including spheroids and organoids, have been established to improve comparability to in vivo situations, differentiation processes, and growth modalities [15,16,17,18]. How far spheroids mimic in vivo lipid metabolism, however, remains poorly understood.

To this end, the primary goal of this work was to evaluate the ability of Caki-1 3D spheroids for studying renal lipotoxicity and lipid accumulation. Caki-1 cells, which have a cancerous origin, have been used as both normal human PTECs and RCC models in vitro [19,20]. We found that Caki-1 cells formed compact spheroids with tight cell-cell contact when grown in a low-attachment environment. We further used the Caki-1 3D culture model to assess lipid accumulation and cellular response under FFA overload.

## 2. Materials and Methods

### 2.1. Caki-1 Cell Line in 2D and 3D Cultures

Caki-1 (human kidney, epithelial, derived from skin metastasis; ATCC HTB46) cell line was purchased from the ATCC (Manassas, VA, USA). For 2D culture, the Caki-1 cell line was maintained in F12/DMEM-normal glucose medium supplemented with 10% fetal bovine serum and 1% penicillin/streptomycin (Fisher Scientific, Hanover Park, IL, USA). The cells were cultured at 37 °C in a humidified 5% CO_2_ incubator. The culture medium was changed every 2 or 3 days. Cells were routinely sub-cultured when 80% confluence was reached using 0.25% *w*/*v* Trypsin-EDTA solution (Fisher Scientific).

For 3D spheroid formation, Caki-1 cells were cultured in Corning microcavity plates with round bottom ultra-low attachment, enabling the formation of a single multicellular spheroid centered in each microwell in a highly reproducible manner. Cells were seeded into the 24-well microplates (Corning Cat. No. 4441) at 1 × 10^5^ cells/well in 1 mL 3D tumorsphere medium XF (Fisher Scientific). To promote spheroid formation, plates were swirled before incubation. Spheroids were then cultured for 10–12 days without medium change.

### 2.2. Fatty Acid Treatment

The monolayer cells were washed with serum-free medium and then treated with saturated palmitate or unsaturated oleate (Sigma-Aldrich Inc., St. Louis, MO, USA) complexed with 0.3–0.5% essential fatty acid-free bovine serum albumin (BSA, A7030, Sigma-Aldrich Inc.) for 24–48 h. Both palmitate and oleate were dissolved in ethanol to make a 300 mM stock solution and then complexed with BSA. All experiments were performed in medium containing 0.25% ethanol. For the 3D culture model, 8 days after cell seeding, spheroids were treated with the indicated fatty acid for 2–4 days. Images were taken before and after adding treatments. Cell lysates were prepared using RIPA lysis buffer with a cocktail of protease inhibitors (Sigma-Aldrich Inc.). The samples were stored at −80 °C for Western blot analysis.

### 2.3. Spheroid Size Evaluation

Pictures for spheroid size evaluation were taken with an Olympus 1X51 inverted microscope with phase-contrast and fluorescence (Olympus America Inc., Center Valley, PA, USA). The spheroid size and shape were evaluated by measuring spheroid perimeter using ImageJ version 1.54 (NIH, Bethesda, MD, USA).

### 2.4. Live/Dead Viability/Cytotoxicity Staining

Analysis of cell viability of Caki-1 3D spheroids was based on fluorescence live/dead staining using the live/dead viability/cytotoxicity kit (L3224B, Fisher Scientific). The kit quickly discriminates live from dead cells by simultaneously staining with green-fluorescent calcein-AM to indicate intracellular esterase activity and red-fluorescent ethidium homodimer-1 to indicate loss of plasma membrane integrity. The incubation of the spheroids was carried out with calcein-AM (2 µM) and ethidium homodimer-1 (4 µM) for 30 min. Analysis was performed using an Olympus 1X51 inverted fluorescence microscope with appropriate filter sets.

### 2.5. Immunofluorescence Staining

Spheroids were fixed with 4% formaldehyde for 30 min and then stained for cleaved caspase-3 (9661, 1:200, Cell Signaling Technology, Danvers, MA, USA). The secondary antibody was Alexa Fluor-conjugated 488 donkey anti-rabbit IgG (1:200) from Jackson ImmunoResearch Laboratories (West Grove, PA, USA). In another set of experiments, BODIPY 493/503 (Fisher Scientific) staining was performed to evaluate intracellular neutral lipids. After nuclear staining with DAPI, the slides were mounted with ProLong gold antifade reagent (Thermo Fisher Scientific, Waltham, MA, USA).

### 2.6. Western Blot Analysis

An equal amount (40 µg) of cell lysates was separated by 10% SDS-PAGE and transferred electrophoretically to a nitrocellulose membrane. The blots were incubated with primary antibodies for rabbit anti-PARP1 (1:3000, Cell Signaling), rabbit anti-LC3B (1:2000, Cell Signaling), rabbit anti-p62 (1:3000, Cell Signaling), rabbit anti-CHOP (1:2000, Cell Signaling), rabbit anti-perilipin 2 (1:3000, Cell Signaling), and mouse anti-β–actin or GAPDH (Sigma-Aldrich Inc.). The secondary antibodies were HRP-conjugated anti-rabbit or anti-mouse IgG (1:6000, Santa Cruz Biotechnology, Dallas, TX, USA). Detection was accomplished using enhanced chemiluminescence Western blotting (ECL, GE Healthcare, Piscataway, NJ, USA). Relative band intensity was measured by densitometry using ImageJ software with β-actin as an internal control.

### 2.7. Statistical Analysis

All analysis was performed using Excel 2022 and GraphPad Prism 10 for Windows (GraphPad Software, La Jolla, CA, USA). Data are expressed as means ± SEM. Multiple-group comparisons were performed by one-way ANOVA and Tukey post hoc test. Statistical significance was set at *p* < 0.05.

## 3. Results

### 3.1. Palmitate-Induced Morphological Change and Cell Death in Caki-1 Spheroids

**Figure 1:**
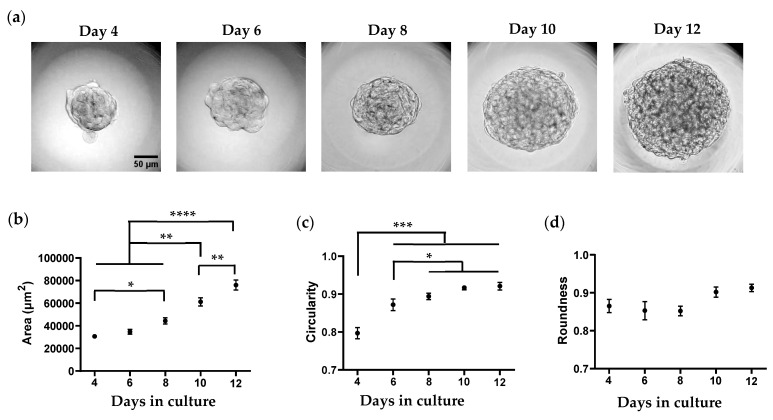
Formation and characterization of Caki-1 spheroids in a 3D culture model. (**a**) Representative images of Caki-1 spheroids captured on days 4, 6, 8, 10, and 12 after seeding. (**b**–**d**) Quantitative analysis of spheroid area (**b**), circularity (**c**), and roundness (**d**) from day 4 to day 12 using ImageJ software. Scale bar: 50 µm. Data represent mean ± SEM. * *p* < 0.05, ** *p* < 0.01, *** *p* < 0.001, and **** *p* < 0.0001. 30 spheroids were analyzed for three independent experiments.

The Caki-1 3D culture model was generated by seeding the cells in 24-well low-attachment microplates. In optimal 3D spheroid-rich conditions, Caki-1 cells formed uniform spheroids with smooth edges. Caki-1 spheroids were imaged and tracked every 2 days, and obtained images were analyzed with ImageJ software, calculating the area of the spheroid along with three shape descriptors: circularity, roundness, and solidity. Circularity indicates the degree of similarity to a perfect circle, a value of 1 indicating perfect. Solidity describes the extent to which a shape is convex or concave, a value of 1 indicating a completely convex shape. Roundness is somewhat like circularity but less sensitive to irregularities along the perimeter and more sensitive to elongated shapes. The representative image (Figure 1a) and the area (Figure 1b) of the spheroids formed in this experiment from day 4 to day 12 demonstrate a gradual increase in area/diameter over time in normal condition. We observed no significant variation in circularity, roundness, and solidity from day 4 to day 12 (Figure 1c,d and Supplementary Figure S1), indicating that these shape descriptors do not reflect changes in spheroid compactness.

**Figure 2:**
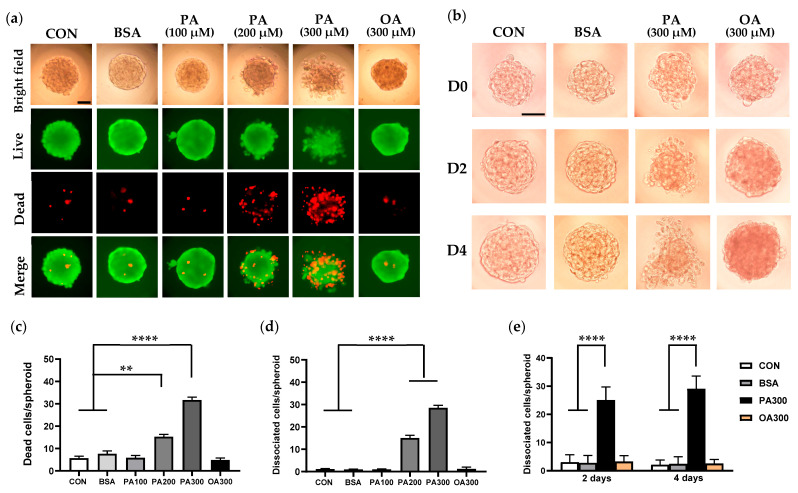
Dose- and time-dependent effects of palmitate on Caki-1 spheroid morphology and cell viability. (**a**) Representative bright-field and live/dead staining images of Caki-1 spheroids following exposure to control (CON), 0.5% bovine serum albumin (BSA) only, or BSA-conjugated palmitate (PA) at concentrations of 100 µM, 200 µM, and 300 µM for 2 days. Live cells were stained in green with calcein AM and dead cells were stained in red with ethidium homodimer I. (**b**) Bright-field images showing time-dependent spheroid disintegration when 8-day-old (D0) spheroids were stimulated with high concentration (300 µM) of PA for 2 (D2) and 4 (D4) days. Scale bar: 50 µm. (**c**,**d**) Quantification of dead (**c**) and dissociated (**d**) cell number in spheroids treated with PA100–300 or OA300 for 2 days. (**e**) Quantification of the number of dissociated cells from the spheroids following fatty acid treatment for 2 and 4 days. Data represent mean ± SEM. ** *p* < 0.01 and **** *p* < 0.0001. 30 spheroids per group were analyzed for three independent experiments.

We have previously reported that treatment with saturated palmitate, but not unsaturated oleate, resulted in significant cell apoptosis in primary mouse tubular cell monolayer [11]. To further evaluate the cytotoxic effect of palmitate on the PT 3D model, Caki-1 spheroids were exposed to multiple concentrations of BSA-conjugated palmitate (100 µM, 200 µM, and 300 µM) after seeding for 8 days. While 100 µM palmitate was chosen as the concentration to represent a normal physiological dietary (serum) level with minimal cell death, 200 and 300 µM palmitate mimic the hyperlipidemic condition in vivo (the situation of diabetes with obesity). A high dose of oleate (300 µM), the most common unsaturated fatty acid in serum and diet, was used for comparison purposes. As depicted in Figure 2a,b, bright-field images showed that palmitate exposure resulted in a dose- and time-dependent spheroid disintegration, whereas there was no apparent morphologic change in spheroids following fatty acid-free BSA (0.3–0.5%) or oleate administration for 2–4 days compared to the normal control. To ascertain whether these phenotypic changes may be a reflection of altered cell survival, we further performed live/dead staining. As illustrated in Figure 2a, individual dead cells were observed in the interior of normal spheroids, which was not different following BSA or low-dose palmitate treatment. Incubation of spheroids with increasing concentrations of palmitate resulted in a dose-dependent cell dissociation and death (Figure 2c,d). The dead cells were robustly increased and distributed throughout the spheroid as the concentration of palmitate increased to 300 µM. Exposure of spheroids to high-dose oleate did not lead to an increase in cell dissociation and death (Figure 2c–e).

### 3.2. PA-Induced Cell Apoptosis in Caki-1 Spheroids

**Figure 3:**
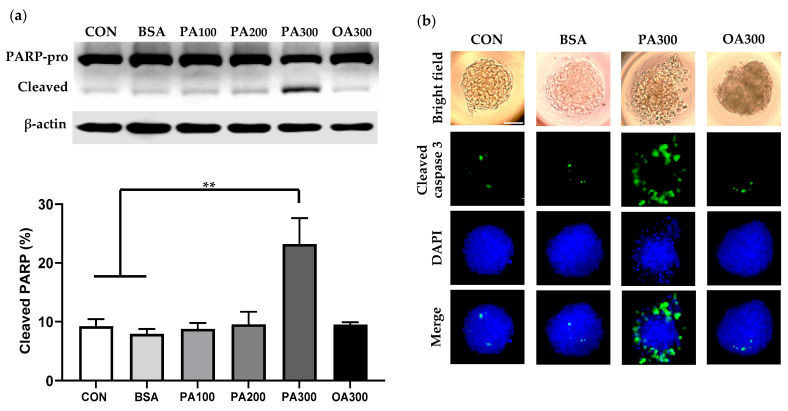
Palmitate-induced apoptosis in Caki-1 spheroids. (**a**) Western blot analysis of full-length PARP (PARP-pro, 116 kDa) and cleaved PARP (86 kDa) in Caki-1 spheroids treated with varying concentrations of palmitate [100 µM (PA100), 200 µM (PA200), and 300 µM (PA300)] or oleate [300 µM (OA300)] for 2 days. Data represent mean ± SEM. *n* = 3; ** *p* < 0.01. (**b**) Immunostaining for active caspase-3 (green) in Caki-1 spheroids following exposure to 300 µM of palmitate or oleate. Blue DAPI stains nuclei. Scale bar: 50 µm.

Next, we performed Western blot and immunostaining to determine cell apoptosis in palmitate-treated spheroids. Palmitate-induced cell apoptosis was first evaluated by Western blot analysis of cleaved poly (ADP-ribose) polymerase (PARP). During caspase-dependent apoptosis, PARP, a nuclear protein, is cleaved by caspases 3 and 7 at its caspase-cleavage site, generating an 89 kDa fragment [21,22,23]. As shown in Figure 3a, palmitate exposure caused a dose-dependent increase in cleaved PARP. Palmitate (300 µM) stimulated the cleavage of PARP to an 89 kDa fragment, whereas PARP fragmentation was not affected by a high concentration of unsaturated oleate. Palmitate-induced cell apoptosis was confirmed by immunostaining for active caspase-3 (Figure 3b). In both normal and BSA controls, very few cleaved caspase-3-positive cells were detected in the middle of spheroids. Palmitate (300 µM) treatment led to a substantial activation of caspase-3 with more staining in dissociated peripheral cells of spheroids, whereas caspase-3 activity was not altered upon oleate stimulation.

### 3.3. Palmitate-Mediated Cytotoxicity Was Blunted by Triacsin C

**Figure 4:**
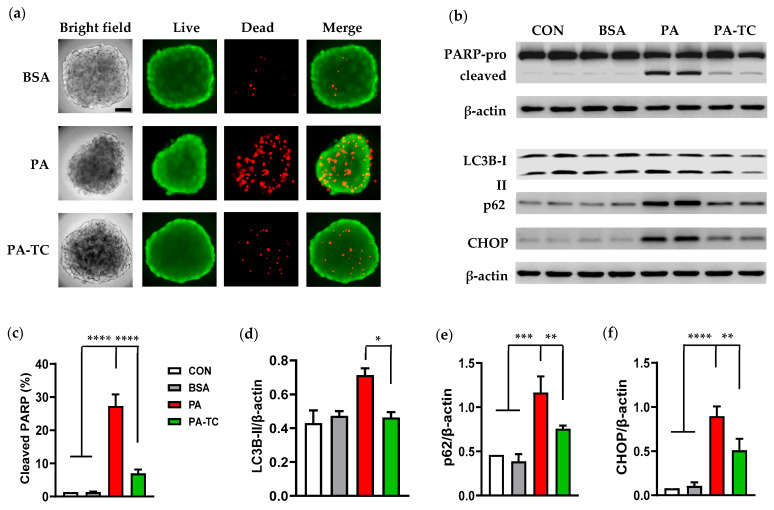
Inhibition of ACSL by triacsin C reduced palmitate-induced cytotoxicity in Caki-1 spheroids. (**a**) Representative live (green) and dead (red) staining images of Caki-1 spheroids treated with triacsin C (4 µM) for 1 h, followed by exposure to palmitate (300 µM) for 48 h. Palmitate (**PA**)-induced cell death was greatly attenuated in the presence of triacsin C (**PA-TC**). Scale bar: 50 µm. (**b**–**f**) Western blot analysis of PARP, LC3B, p62, and CHOP proteins in Caki-1 spheroids under the same treatment conditions. Palmitate-induced PARP cleavage (**c**) and upregulation of autophagy and ER stress markers LC3B-II (**d**), p62 (**e**), and CHOP (**f**) were significantly ameliorated by triacsin C. Data represent mean ± SEM. n = 3–4; * *p* < 0.05, ** *p* < 0.01, *** *p* < 0.001, and **** *p* < 0.0001.

Palmitate metabolism depends on its conversion to palmitoyl-CoA, which is then incorporated into downstream metabolites such as PL, DAG, and TAG [24]. Such incorporations depend on long-chain acyl-CoA synthetase (ACSL) to first convert palmitate into palmitoyl-CoA, and ACSL can be inhibited by triacsin C to prevent palmitate metabolism [24]. We next determined if palmitate metabolism is required for its cytotoxicity in Caki-1 spheroids following ACSL inhibition by triacsin C. To this end, Caki-1 spheroids were pre-treated with triacsin C (4 µM) for 1 h, followed by co-culture with triacsin C and palmitate (300 µM) for 48 h. As depicted in Figure 4a, live/dead staining showed that palmitate-induced cell death was greatly attenuated in the presence of triacsin C. Accordingly, triacsin C pre-incubation significantly inhibited palmitate-stimulated PARP cleavage (Figure 4b,c).

Since defective autophagy has been linked to lipotoxicity in several cellular models, we next investigated if autophagy activity was altered in palmitate-treated Caki-1 spheroids by evaluating the expression level of the two commonly used autophagy markers, LC3-II and SQSTM1/p62. We found that 48 h treatment of Caki-1 spheroids with palmitate significantly upregulated SQSTM1/p62 (Figure 4b,e), indicating an inhibition of autophagosome degradation by palmitate. In addition, an upregulation of CHOP, a protein related to unfolded protein response, confirmed palmitate-induced ER stress in Caki-1 spheroids. As depicted in Figure 4b,f, triacsin C treatment prevented palmitate-induced autophagy impairment and ER stress, as evidenced by a reduction in p62 and CHOP protein levels. These results support that ACSL-dependent palmitate metabolism is required for its detrimental effects on renal PTECs.

### 3.4. Oleate Ameliorated PA-Mediated Cytotoxicity in Caki-1 Spheroids

**Figure 5:**
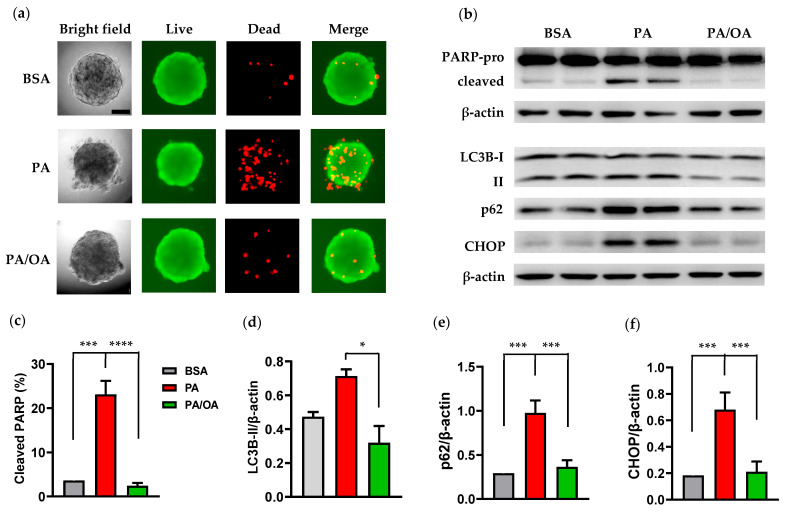
Oleate co-incubation protected against palmitate-induced cytotoxicity in Caki-1 spheroids. (**a**) Representative live (green) and dead (red) staining images of Caki-1 spheroids treated with **BSA**, palmitate (**PA**, 300 µM), or palmitate (300 µM) plus oleate (150 µM) (**PA/OA**) for 48 h. Oleate co-incubation substantially reduced the number of dead cells. Scale bar: 50 µm. (**b**–**f**) Western blot analysis of cleaved PARP, LC3B, p62, and CHOP in Caki-1 spheroids under the same treatment conditions. Oleate co-incubation greatly suppressed palmitate-induced PARP cleavage (**c**) and upregulation of autophagy and ER stress markers LC3B-II (**d**), p62 (**e**), and CHOP (**f**). Data represent mean ± SEM. *n* = 3–4; * *p* < 0.05, *** *p* < 0.001, and **** *p* < 0.0001.

Co-treatment with unsaturated oleate has been shown to ameliorate palmitate-induced apoptosis in proximal tubular cells by enhancing intracellular LD formation [25]. Our preliminary study showed that simultaneous treatment with oleate (50–300 µM) prevented palmitate (300 µM)-mediated PARP cleavage in Caki-1 2D culture (Supplementary Figure S2). To further determine if oleate co-incubation was also protective in Caki-1 3D culture, the spheroids were stimulated with 300 µM palmitate in the presence of 150 µM oleate. Spheroids co-treated with oleate maintained better structural integrity and had reduced cell death compared to those treated with palmitate alone (Figure 5a). Moreover, oleate prevented PARP cleavage and reduced p62 and CHOP protein expression in palmitate-overloaded spheroids (Figure 5b–f). These results suggest that oleate can counteract the cytotoxic effects of palmitate by restoring autophagy function and inhibiting ER stress in the Caki-1 3D model.

### 3.5. FFA Overload Induced Perilipin 2 Expression and LD Formation

**Figure 6:**
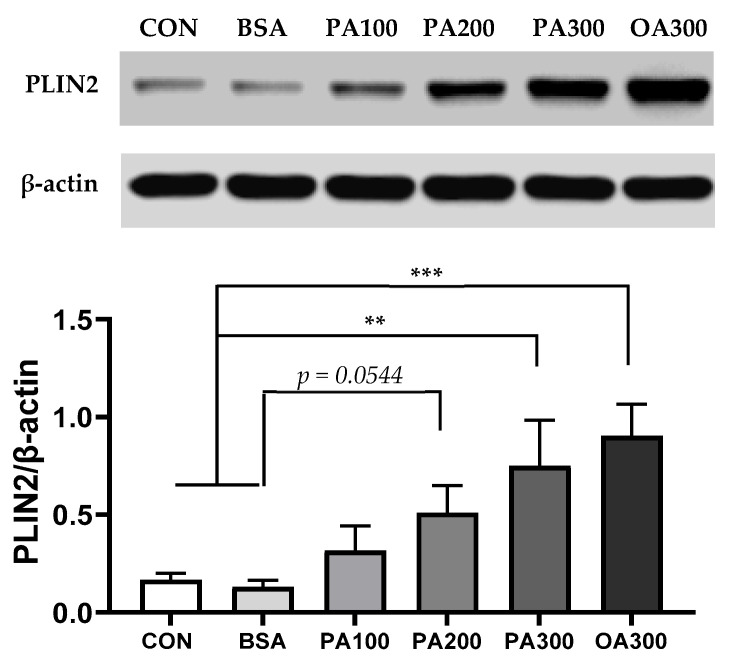
Fatty acid treatment induced PLIN2 expression in Caki-1 spheroids. Western blot analysis showing a significant upregulation of PLIN2 expression in spheroids treated with high doses of palmitate and oleate compared to low-dose and control conditions. Data represent mean ± SEM. *n* = 3–4; ** *p* < 0.01 and *** *p* < 0.001.

As hubs of lipid metabolism, LDs provide a dynamic lipid repository that can safely sequester lipids to prevent lipotoxicity and can be mobilized upon cellular demand to provide substrates for the generation of energy (i.e., β-oxidation) or the biosynthesis of lipid signaling molecules and membranes. The LD core consists of triglycerides (TGs) and sterol-esters surrounded by a phospholipid monolayer and surface proteins such as perilipin 2 [PLIN2, also known as adipose differentiation-related protein (ADRP)]. To evaluate the effects of FFA treatment on LD formation in Caki-1 spheroids, PLIN2 protein, a marker for lipid droplets, was quantified by Western blot. Both palmitate and oleate significantly upregulated PLIN2 expression compared to normal and BSA controls (Figure 6). BODIPY 493/503 stain was also performed to visualize neutral lipids in spheroids following FFA treatment for 48 h. As depicted in Supplementary Figure S3, bright lipid vesicles were observed in oleate-treated spheroids.

### 3.6. Knockdown of PLIN2/ADRP Enhanced Apoptotic Cell Death in Caki-1 Cells

**Figure 7:**
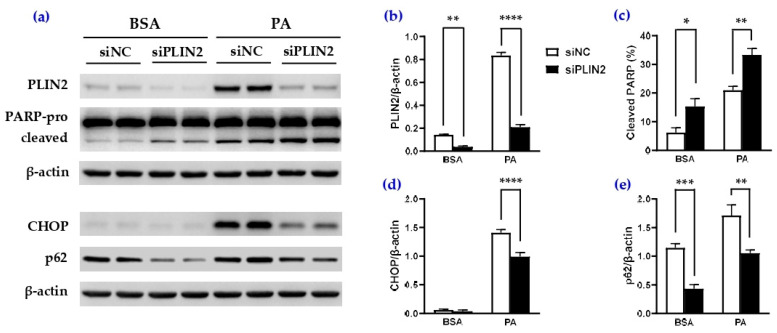
PLIN2 knockdown enhanced PARP cleavage in BSA and palmitate-stimulated Caki-1 cells. (**a**) Representative Western blot images of PLIN2, PARP, CHOP, and p62 proteins in Caki-1 cells treated with BSA or palmitate (PA, 300 µM) for 24 h after transfection with negative siRNA (siNC) or siRNA targeting PLIN2/ADRP (siPLIN2). (**b**–**e**) Quantitative analysis of PLIN2 (**b**), cleaved PARP (**c**), CHOP (**d**), and p62 (**e**) proteins in BSA or PA-treated cells. Data represent mean ± SEM. *n* = 3–4; * *p* < 0.05, ** *p* < 0.01, *** *p* < 0.001, and **** *p* < 0.0001.

As a constitutively expressed LD protein, PLIN2 is also required for the formation and stability of the LD organelle [26,27]. Previous studies have indicated a role of PLIN2 in modulating autophagy, ER stress resolution, and β cell survival and function [28,29]. To examine the significance of PLIN2 upregulation in palmitate-mediated lipotoxicity, we performed siRNA transfection to knock down PLIN2 (siPLIN2) in Caki-1 cells. Compared to negative siRNA (siNC) transfection, Western blot analysis confirmed that siPLIN2 transfection led to a ~80% decrease in PLIN2 in BSA-treated cells and greatly inhibited palmitate-induced PLIN2 upregulation (Figure 7a,b). The downregulation of PLIN2 resulted in an increase in cleaved PARP in BSA-treated cells (Figure 7a,c). Moreover, siPLIN2 significantly enhanced palmitate-stimulated PARP fragmentation, indicating an exacerbation of apoptotic cell death by PLIN2 deficiency. Palmitate-mediated CHOP and p62 upregulation was suppressed by siPLIN2 (Figure 7a,d,e). These results suggest that PLIN2 expression exhibits a cytoprotective role under basal conditions and upon palmitate overload.

## 4. Discussion

The globally increasing prevalence of kidney diseases and difficulty of treating RCC combined with the obesity pandemic highlight the need to better understand the role of metabolic disease and lipids in these diseases. In the present study, we sought to evaluate FFA-mediated lipotoxic responses in a 3D culture model. Using Caki-1 spheroids, we confirmed that overload with saturated palmitate induced a dose- and time-dependent spheroid disintegration and apoptotic cell death. Palmitate-induced cytotoxic effects were prevented by preincubation with triacsin C to inhibit lipid metabolism or the addition of unsaturated oleate to promote LD formation. Additionally, we found that the knockdown of LD protein PLIN2 exacerbated apoptotic cell death in Caki-1 cells.

In optimal 3D culture conditions, Caki-1 cells formed well-defined, large, compact spheroids with uniform morphology, good circularity, and increased diameter from days 4–12. Spheroids treated with BSA only maintained intact round structures with individual cell death in the middle of spheroids, confirming that BSA itself was not toxic in 3D culture. However, spheroids incubated with palmitate exhibited irregularly dispersed morphology with increased dead cells in the peripheral layers and diminished spheroid core, which was not observed following oleate activation. Our results support that Caki-1 spheroids can serve as a simple and reliable 3D culture model to evaluate FFA-induced lipotoxicity.

This study also confirmed lipotoxicity-induced apoptosis as evidenced by an increase in PARP fragmentation and caspase-3 activity in palmitate-treated spheroids. PARP is a substrate of activated caspases-3 and 7 in caspase-dependent apoptosis. During caspase-dependent apoptosis, caspases-3 and 7 cleave PARP at its specific caspase-cleavage site, producing a 24 kDa fragment containing the DNA binding domain and an 89 kDa fragment containing the catalytic and automodification domains [30,31]. After PARP cleavage by caspase, the 24 kDa PARP1 fragment irreversibly binds to DNA breaks and acts as a transdominant inhibitor of active PARP, whereas the 89 kDa PARP fragment is translocated to the cytoplasm [31]. Thereby, PARP fragmentation by caspase leads to its inactivation, which inhibits DNA repair and facilitates caspase-mediated DNA fragmentation in apoptosis. An increase in 89 kDa PARP fragment confirmed palmitate-induced apoptosis in Caki-1 spheroids. Immunostaining spheroids for active caspase-3 further revealed a substantial increase in caspase-3 activity at the external layers of the spheroids, suggesting that the dissociated cells underwent apoptosis upon palmitate overload.

Palmitate is known to cause cell apoptosis through different mechanisms, including its activation to palmitoyl-CoA [32,33]. In the current study, we found that pretreatment of spheroids with triacsin C, an inhibitor of ACSLs, blunted palmitate-mediated cell death and PARP fragmentation. Moreover, triacsin C administration suppressed PA-stimulated ER stress and restored autophagy function by reducing the protein levels of CHOP and p62. Our results suggest that ACSL-dependent fatty acid activation mediates the cytotoxic effects of palmitate in Caki-1 spheroids. However, this study did not determine the specific ACSL family member(s) contributing to palmitate metabolism and lipotoxicity since triacsin C acts as a competitive inhibitor of multiple ACSL subtypes [34]. Further investigation about the role of each ACSL isotype in renal lipid metabolism and the detailed underlying mechanisms is warranted.

In line with the findings in PTEC 2D monolayer that oleate does not cause cell dysfunction and apoptosis [10], Caki-1 spheroid morphology and cell viability were not affected by oleate treatment. We also observed that co-incubation of spheroids with oleate led to a suppression of palmitate-induced cytotoxic effects. Increased LD formation has been shown to correlate positively with the protective effects of unsaturated FFAs and negatively with cell damage in both cell culture and mouse kidneys [10]. Consistent with the findings in other cell types [14,35,36,37], oleate overload promoted more abundant and bigger LD accumulation in Caki-1 cells than palmitate. During palmitate-induced lipotoxic stress, cell viability is mainly determined by mitochondrial and ER homeostasis [10]. LDs participate in controlling energy metabolism and can function as a reservoir for unsaturated lipids to prevent ER stress [10,14,38]. Increased LD formation by oleate may promote the capacity to buffer the lipotoxic stress in the ER and thus improve autophagy activity, which was reflected by a reduction in CHOP and p62 in Caki-1 spheroids co-treated with oleate and palmitate.

Abnormal accumulation of LDs with PLIN2 localization in the kidney tubules has been reported in DKD patients [39,40] and animal models [41]. In this study, we confirmed an increase in PLIN2 upon palmitate and/or oleate stimulation in Caki-1 spheroids. PLIN2 downregulation significantly increased PARP cleavage in BSA-treated cells upon serum deprivation, suggesting an important role for PLIN2 in PTEC survival. An acceleration of palmitate-induced PARP cleavage in siPLIN2-transfected cells provides further evidence supporting a cytoprotective role for PLIN2 against saturated FFA-mediated cell toxicity. This study also revealed a reduction in CHOP and p62 proteins by siPLIN2, indicating that PLIN2 knockdown led to an attenuation of ER stress response in Caki-1 cells. Although our results agree with the previous reports that PLIN2 downregulation was associated with enhanced autophagic flux and accelerated ER stress resolution in β cells [28] and hepatocytes [42], it is important to note that the overexpression of PLIN2 has been shown to ameliorate palmitate-induced ER stress and prevent against cell apoptosis in murine proximal tubular (mProx24) cells [4]. We speculate that there are different mechanisms underlying PLIN2 overexpression and deficiency-associated reduction in ER stress when the cells are exposed to excess saturated palmitate. Ectopic overexpression of PLIN2 in PTECs may promote the formation of LDs, which act as buffers to sequester misfolded proteins and excess lipids and then alleviate lipotoxic ER stress [43,44]. Following PLIN2 knockdown, a decrease in p62 suggests increased lysosomal degradation due to the improvement of autophagy flux, which may result in the more effective resolution of ER stress [42]. However, PLIN downregulation-associated decrease in p62 and CHOP did not lead to an attenuation of lipotoxicity. Instead, enhanced apoptotic cell death was observed in siPLIN2-treated Caki-1 cells. Additional studies will be necessary to understand the regulation and functional role of PLIN2 in lipid intracellular trafficking and utilization under nutritional stress.

Taken together, our results suggest that the 3D Caki-1 spheroid model is a reproducible in vitro system allowing for the simple assessment of cellular cytotoxicity in response to different FFA stimulations. Caki-1 cells are used as both models of RCC and PT, although with limitations with regards to the expression patterns of PT markers and transport proteins relative to freshly isolated primary cells [45]. Despite these caveats, our studies illustrate the utility of Caki-1 spheroids to enhance the understanding of the under-studied yet increasingly important topic of lipotoxicity in the kidney.

## Data Availability

The original contributions presented in this study are included in the article/Supplementary Materials. Further inquiries can be directed to the corresponding author.

## Supplementary Materials

The following supporting information can be downloaded at: https://www.mdpi.com/article/10.3390/cells14050349/s1, Figure S1: Morphological analysis of Caki-1 3D spheroids; Figure S2: Effects of oleate on palmitate-induced PARP cleavage in Caki-1 2D culture; Figure S3: Lipid staining by BODIPY in FFA-treated Caki-1 spheroids.
