# Supplementary material for: Caki-1 Spheroids as a Renal Model for Studying Free Fatty Acid-Induced Lipotoxicity
Source: Cells. 2025 Feb 27;14(5):349. doi: 10.3390/cells14050349 (PMC11899473; doi:10.3390/cells14050349)

Supplementary Figure Legends

Supplementary Figure S1. Morphological analysis of Caki-1 3D spheroids. Quantitative analysis of spheroid solidity on days 4–12 after seeding. Data represent mean ± SEM. **** $p < 0.0001$. 30 spheroids were analyzed for three independent experiments.

Supplementary Figure S2. Effects of oleate on palmitate-induced PARP cleavage in Caki-1 2D culture. 80–90% confluent Caki-1 cells were treated with BSA, 300 μM of palmitate (PA300) in the absence or presence of oleate 50 μM (OA50), 100 μM (OA100), 200 μM (OA200), 300 μM (OA300) for 24 h. Representative Western blot images showed that palmitate-induced PARP cleavage was inhibited by oleate co-administration.

Supplementary Figure S3. Lipid staining by BODIPY in FFA-treated Caki-1 spheroids. Intracellular neutral lipid (green) staining by BODIPY 493/503 in Caki-1 spheroids treated with 300 μM of palmitate (PA300) or oleate (OA300). Blue DAPI stains nuclei. Scale bar: 50 μm.

Supplementary Figure S1. Morphological analysis of Caki-1 spheroids.

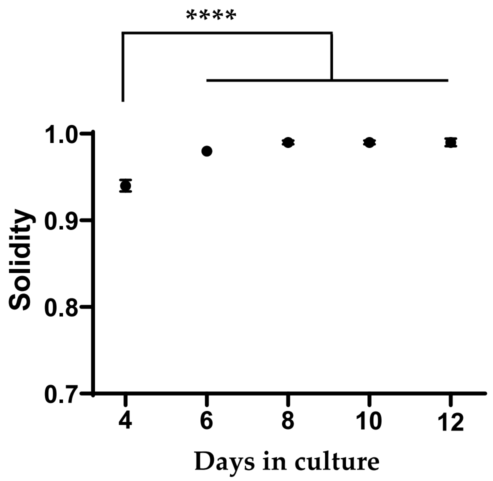

Supplementary Figure S2. Effects of oleate on palmitate-induced PARP cleavage in Caki-1 2D culture.

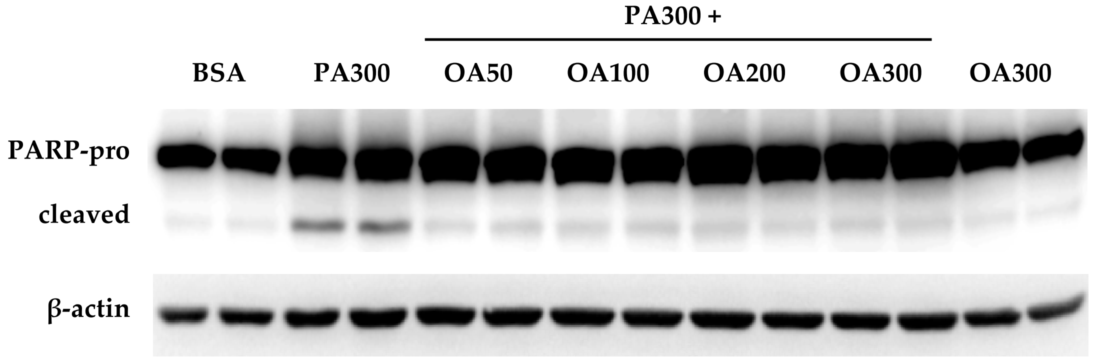

Supplementary Figure S3. Lipid staining by BODIPY in FFA-treated Caki-1 spheroids.

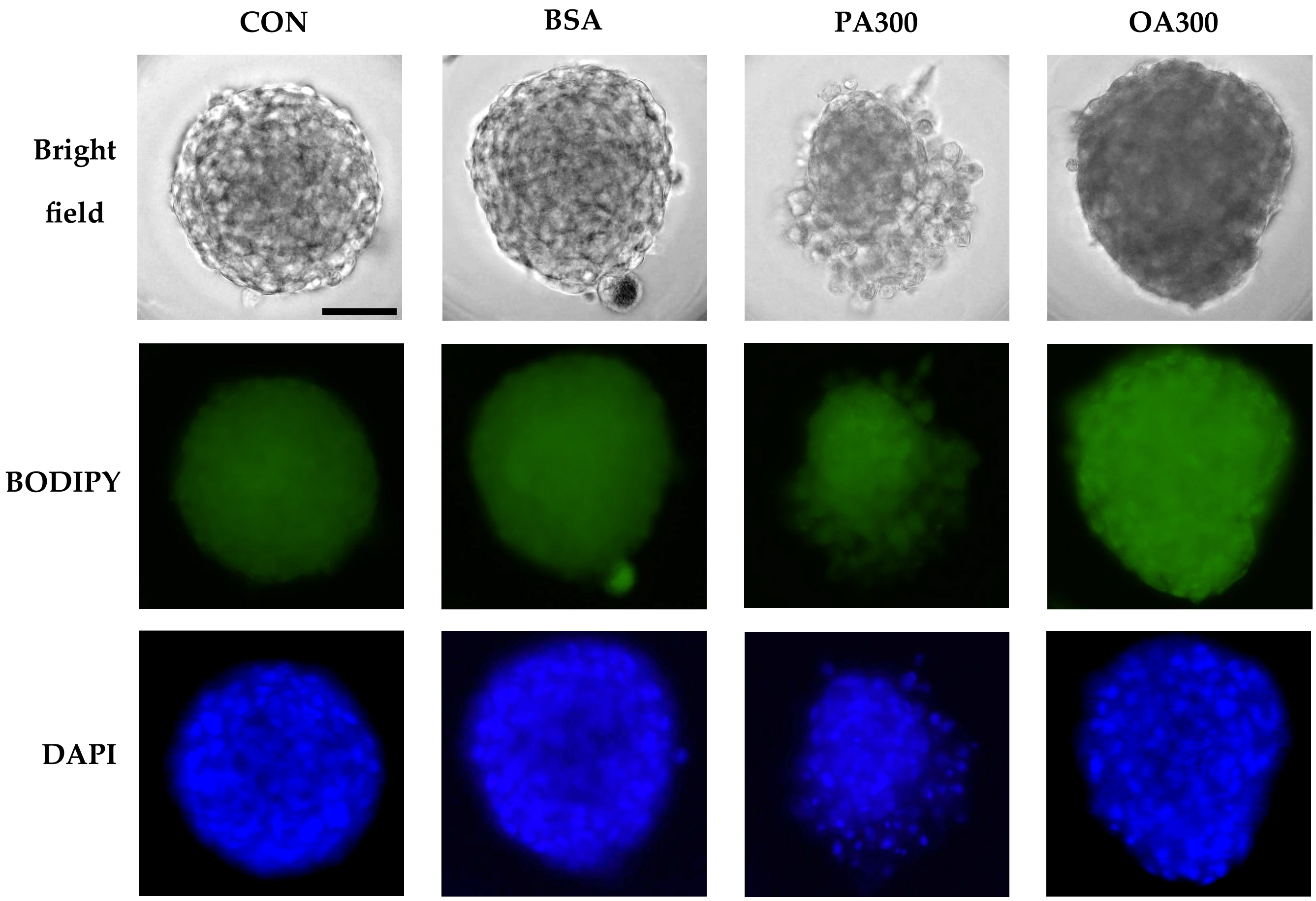

Supplement: Supplementary file 1 [file cells-14-00349-s001.zip › cells-3412497-supplementary.pdf]
